# Zebrafish: A Model for the Study of Toxicants Affecting Muscle Development and Function

**DOI:** 10.3390/ijms17111941

**Published:** 2016-11-19

**Authors:** Magda Dubińska-Magiera, Małgorzata Daczewska, Anna Lewicka, Marta Migocka-Patrzałek, Joanna Niedbalska-Tarnowska, Krzysztof Jagla

**Affiliations:** 1 Department of Animal Developmental Biology, Institute of Experimental Biology, University of Wroclaw, 21 Sienkiewicza Street, 50-335 Wroclaw, Poland; magda.dubinska-magiera@uwr.edu.pl (M.D.-M.); anna.lewicka@uwr.edu.pl (A.L.); marta.migocka-patrzalek@uwr.edu.pl (M.M.-P.); joanna.niedbalska-tarnowska@uwr.edu.pl (J.N.-T.); 2 GReD—Genetics, Reproduction and Development Laboratory, INSERM U1103, CNRS UMR6293, University of Clermont-Auvergne, 28 Place Henri-Dunant, 63000 Clermont-Ferrand, France

**Keywords:** zebrafish, skeletal muscles, toxicants, biosensors, pollutants

## Abstract

The rapid progress in medicine, agriculture, and allied sciences has enabled the development of a large amount of potentially useful bioactive compounds, such as drugs and pesticides. However, there is another side of this phenomenon, which includes side effects and environmental pollution. To avoid or minimize the uncontrollable consequences of using the newly developed compounds, researchers seek a quick and effective means of their evaluation. In achieving this goal, the zebrafish (*Danio rerio*) has proven to be a highly useful tool, mostly because of its fast growth and development, as well as the ability to absorb the molecules diluted in water through its skin and gills. In this review, we focus on the reports concerning the application of zebrafish as a model for assessing the impact of toxicants on skeletal muscles, which share many structural and functional similarities among vertebrates, including zebrafish and humans.

## 1. Introduction

Many different lines of evidence have revealed that the zebrafish is an excellent model to study various aspects of developmental biology and molecular genetics. In the recent past, the zebrafish was also used as a model for toxicological and drug discovery research which were described in excellent reviews [1,2,3]. However, our paper is focused on toxicants influence on muscle tissue functions and development. As an alternative to mammalian model species, zebrafish is distinguished by numerous advantages, e.g., adults produce a high number of externally developing embryos, husbandry is cheaper than mice, and individuals grow at a fast rate (mature adults develop in about three months). Moreover, embryos are relatively large and initially transparent, which allows real-time imaging of all developmental stages [4]. Of interest, zebrafish embryo testing is classified as in vitro testing and is not subject to EU legislation covering the use of animals for scientific purposes [5]. The most important feature of zebrafish as a model is the fact that its genome has been fully sequenced. A body of evidence reveals that 71.4% of human genes are related to zebrafish genes [6]. Analysis of mapping data showed conservation between zebrafish and human genes and revealed significant degrees of synteny between conserved genes [7,8]. It has also been shown that major zebrafish tissues and organs share many features with human counterparts at the anatomical, physiological and molecular levels, including brain, heart, muscles, kidney, and liver [9,10,11,12,13]. Even though most of the toxicological tests are performed on zebrafish embryos, the adult organism is also an excellent model to study toxicants influence on mature organism physiology, lifespan, disease and behavior [14,15,16,17]. It has been demonstrated that adult zebrafish is a suitable model to measure the effects of the exposure to variety of chemicals including therapeutic drugs and environmental toxicants [16,17]. However, the results obtained on adults zebrafish can not be directly compared to those obtained on embryos because different developmental periods appeared to be differentially sensitive to the same compound [18].

It is also worth noting that the toxicity of drugs and environmental pollutants has been shown to be well conserved between humans and zebrafish [19,20,21]. The permeability to many chemicals and drugs makes zebrafish embryos and larvae an accurate model for large-scale drug screening for teratogenicity, cardiotoxicity, neurotoxicity, hepatotoxicity, and nephrotoxicity [22,23,24,25,26]. Zebrafish larvae absorb small molecules diluted in water through their skin and gills, whereas large molecules and proteins can be microinjected to the yolk sac, the sinus venosus or circulation system. Drugs also enter the zebrafish body orally at the protruding-mouth stage (72 hpf) [27]. Due to the small size of the animal, the costs of tested reagents and toxicants can be reduced. It has also been reported that zebrafish mutants are frequently useful to identify specific genes affected by exposure to various toxicants. For example, mutants with an affected cardiovascular system such as *silent heart*, *polka*, *breakdance*, and *hiphop* [28], or embryos with heart abnormalities exposed to toxicants [29,30], survive to later embryonic stages than rodents. This facilitates investigating the impact of toxicants at the molecular level and finding pathways that attenuate toxicity.

Despite numerous zebrafish advantages, there are several limitations of this model organism. It has been reported that zebrafish underwent whole-genome duplication events during teleost evolution. Some of the duplicated genes are no longer expressed in the same tissue as the orthologs, while the others possess new functions. Therefore, mutations in zebrafish paralogs may show a less severe phenotype [4]. Other disadvantages include a limited number of antibodies specific for zebrafish and the costs associated with starting a zebrafish facility [31]. Zebrafish as a model used to monitor the toxicity of environmental contaminants possess some disadvantages such as low sensitivity and inconvenient statistical experiments. Since fish embryos have the protective envelope, named a chorion, the diffusion of certain chemicals, e.g., ethanol, is limited [32].

Because the skeletal muscles in zebrafish comprise about 60% of adult body mass, studies on toxic effects on muscles are of high relevance [33]. The trunk muscles in adult zebrafish are subdivided into approximately 32 segments (called myotomes) along the anterior-posterior axis. Dorsoventrally each myotome is subdivided by the connective tissue horizontal septum into epaxial muscles, which occupy the dorsal part of the myotome, while hypaxial muscles are located ventrally. Within the myotome two types of muscles fibers can be distinguished: red (slow) muscles found just underneath the skin and white (fast) muscles located internally [34,35,36]. Red muscle fibers contain more mitochondria and myoglobin. They are dependent on aerobic metabolism and perform slow and sustained contractions for a prolonged period without fatigue. In contrast, white muscle fibers are characterized by fast contractions, anaerobic metabolism, less myoglobin, and fewer mitochondria. They react fast, with brief, forceful contractions, and are sensitive to extended effort. In both fish and mammals, red and white muscles are characterized by similar anatomy and physiology [37]. In zebrafish, individual muscle fibers are polyneuronally innervated by single primary and one or more secondary motor neurons [38]. Both tissues form a physical connection called a neuromuscular junction (NMJ) ensuring motor neuron-triggered contractile muscle activity.

Receptor over-stimulation and the associated increase of calcium level in muscle are the hypothetical cause of the myopathy via excitotoxic mechanisms [39,40,41].

Skeletal muscles in zebrafish originate from the paraxial mesoderm, which undergoes segmentation into repetitive units called somites [42]. During early embryonic development somites differentiate into three compartments: the dermomyotome, myotome, and sclerotome. Only the myotome is a source of skeletal muscles of the trunk and pelvic fin muscles [43,44]. Like in mammals, fully developed zebrafish trunk muscles are cylindrical, multinucleated muscle fibers. When compared to higher vertebrates (birds and mammals) trunk muscle differentiation in zebrafish is initiated at an earlier stage of development, which could be explained by external fertilization and rapid ability to swim. In zebrafish, myogenesis starts in the unsegmented mesoderm, where slow muscle precursors originate from adaxial cells laterally covering the notochord. Then adaxial cells migrate laterally from the notochord, where they differentiate into the red muscles and take a dorso-ventral position in the myotome. Following the movement of slow muscle precursors to the surface of the myotome, deeper cells differentiate into white muscle fibers [36,45]. In mammals and zebrafish muscles grow through two mechanisms: hypertrophy (enlargement of existing fibers) and hyperplasia (recruitment of new fibers) [46,47,48,49,50]. It is now clearly established that a common genetic program ensures formation of multinucleate muscle fibers in mammals and in zebrafish. In particular, specification and differentiation of skeletal muscles remain under the control of four evolutionarily conserved basic helix-loop-helix (bHLH) myogenic regulatory factors (MRFs) including MyoD, Myf-5, MRF-4 and myogenin. Spatiotemporal expression of individual MRFs could, however, differ between species, affecting their redundant versus specific roles during muscle differentiation [51].

In this review, we discuss the knowledge based on original papers available in scientific databases. In Table 1 we summarize how the zebrafish model could be applied in assessing the impact of toxicants and bioactive compounds on neuromuscular system development and functions.

## 2. Biosensors and Their Applications for Toxicological Research

**Figure 1:**
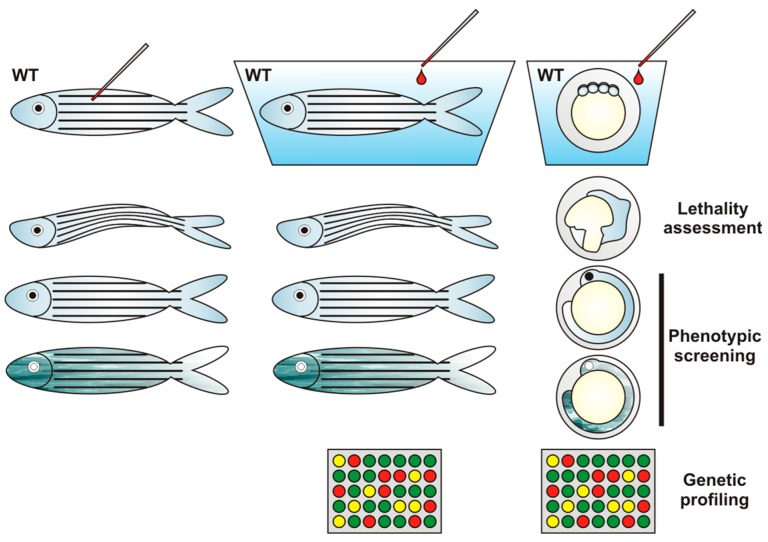
Zebrafish as a tool for toxicological studies. In classical toxicology research, an excessive or non-excessive (e.g., environmentally relevant) dose of the tested compound is administered to an animal e.g., via injection, dietary or waterborne uptake. Evaluation of tested chemical toxicity can be based on various approaches such as lethality assessment, phenotypic screening or gene profiling. Phenotypic screening involves the monitoring of different parameters, e.g., endocrine disrupting compounds (EDCs) can be identified via gonadal morphology and histological comparative analysis. Gene profiling is used in so-called toxicogenomics due to the organism’s susceptibility to different chemicals manifested in the induction of genes, e.g., involved in detoxification or protection against cellular stresses. This method of toxicant identification consists of the assessment of changes in gene-expression profiles by the use of oligonucleotide microarray. Of note, the sensitivity of this assay system is high enough to detect a distinct compound at a concentration that does not cause morphological effects. WT, wild type.

Classical toxicology has its limitations, particularly for assessment of environmental pollutants. It is based on procedures in which an excessive dose of the tested compound is administered to an animal e.g., via injection, in a way that does not occur in the lifespan of an organism, in contrast to dietary or waterborne uptake that occurs naturally (Figure 1).

Detection of a specific toxicant can be indirect, based on the monitoring of different parameters. For example, toxic heavy metals can be identified via monitoring enzymes activity or biomarker gene expression [82], whereas for assessment of endocrine disrupting compounds (EDCs), gonadal morphology and histological comparative analysis, mRNA and protein levels of chorionic gonadotropin or vitellogenin (vtg) are used [83,84,85]. These assays can be conducted in both adult zebrafish and in embryos [86].

Recently, it was proven that zebrafish fertilized eggs can also be used as a bioindicator for water pollution via lethality assessment in conformity to the international organization for standardization (ISO) methodology [86]. In this qualitative assay, the number of dead eggs or embryos and other pollutant-induced disturbances are scored.

Early developmental stages of vertebrate embryos are susceptible to different exogenous chemicals, leading to the induction of genes involved in detoxification or protection against cellular stresses, facilitating adaptation to unfavorable environmental conditions [87].

Zebrafish allows for large-scale analysis of the effects of chemicals on the developing embryos via assessment of gene-expression profiles using oligonucleotide microarray or RNAseq approaches. The toxicogenomic response of the zebrafish embryo is highly sensitive and tissue-specific with consideration of developmental stage. The sensitivity of this assay system is high enough to detect a particular compound at a concentration that does not cause morphological effects. On the one hand, it allows one to determine the transcriptional profile induced by a specific compound and, on the other hand, its application enables identification of the specific compound from the expression profile with high probability [88].

Toxicogenomics seems to be an attractive alternative to traditional methods based on morphological assessment, mostly due to its relatively low costs and reliability, which enables large-scale screening of a wide range of chemicals [89].

Another important issue in toxicological studies is the dosage of the tested compound. In traditional toxicological methods, toxicants are tested by exposing the animal to doses higher than those found in the environment. Thereby, results obtained in this way may be flawed because the typically used extrapolation from high to low doses is not always linear [90].

Difficult to study but very important issue is the long-term impact of toxicants, which includes the effect induced by multi-generational exposure. Using zebrafish, a vertebrate model with a short generation time, for toxicological research enables investigation of chronic exposure in both the parental generation and its offspring [67].

**Figure 2:**
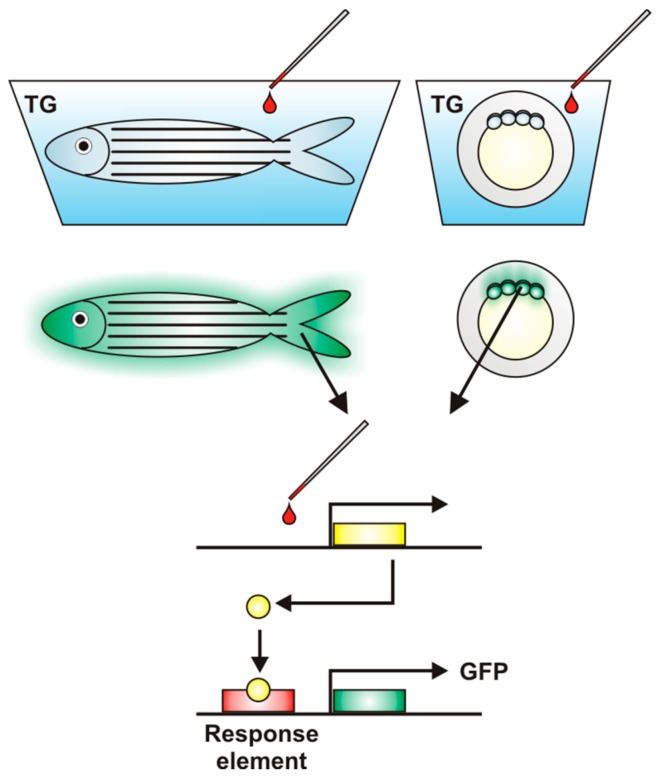
Transgenic (TG) zebrafish as a biosensor for toxicant identification. The modern toxicological approach takes advantage of biotechnological techniques, which allow the development of various zebrafish transgenic lines equipped with the reporter genes such as green fluorescent protein (GFP). Induction of reporter gene expression is driven by specific response elements providing the possibility for demonstration of the tissue-specific mode of toxicant action. These methods have improved the sensitivity and effectiveness of detection in comparison to traditional toxicological techniques. These types of genetically modified zebrafish lines are excellent biosensors and are used for precise qualitative and quantitative analysis of a wide range of potential toxicants.

Biotechnological techniques allow one to avoid many of these problems through the generation of zebrafish transgenic lines carrying reporter genes such as green fluorescent protein (GFP), a way to improve the sensitivity and efficacy of detection of various toxicants [91] (Figure 2). These genetically modified lines play the role of excellent biosensors which, due to the use of specific genetic regulatory elements to drive the expression of fluorescent proteins, provide both the possibility for demonstration of the tissue-specific mode of toxicants’ action and the presence of target classes of pollutants [88,92,93]. Moreover, biosensors enable assessment of different compounds’ toxicity at relatively low doses corresponding to ecologically relevant concentrations.

Recently, an in vivo biosensor system with a quantitative readout for assessment of toxicants’ influence on motor function was successfully developed [93]. This transgenic zebrafish line *TgBAC* (*hspb11:GFP*) expresses a GFP reporter under the control of regulatory elements of the small heat shock protein *hspb11*, which is up-regulated specifically by chemicals that interfere with motor function [94]. In addition, toxicants causing motility defects trigger reversible and dose-dependent *hspb11* transgene expression accompanied by changes in the level of GFP intensity. The *hspb11* up-regulation response upon over-excitation of the muscle causing its hyperactivity is associated with the increase of intracellular Ca^2+^ levels in the muscle cells. These features make the *TgBAC* (*hspb11:GFP*) zebrafish line an excellent model system for quantitative evaluation of toxicant-induced muscle hyperactivity.

It is worth mentioning that transgenic lines appropriate for motor behavior impairments associated with the abnormal development of motor neurons and neuromuscular junctions, caused by different chemicals have been established [95,96,97].

The approach involving identification and application of novel response elements determines the direction for the development of modern biosensors intended for assessment of the toxicity of manmade compounds and monitoring environmental pollution. Tools such as the zebrafish gene chip technology will improve the efficiency of this approach and help in the design of zebrafish transgenic lines. These types of lines will be used for precise qualitative and quantitative analysis of a wide range of chemicals and enable their simultaneous detection [3].

## 3. Pollutants

Over the past years, researchers have demonstrated that zebrafish is well suited for monitoring environmental contamination. Moreover, advances in genetic engineering allow the creation of transgenic zebrafish lines with improved detection sensitivity, which play a role as bioindicators (see above). A broad range of features can be assessed and measured during environmental monitoring. They include changes in morphology, physiology, behavior, and gene expression. Contaminants, which can be detected using a zebrafish model system, include heavy metals and organic pollutants such as endocrine disruptor compounds. Many of these affect the performance and integrity of the skeletal muscle—the tissue that constitutes the bulk of the soft tissues in animals. Due to the limited availability of research tools, their mode of action still remains elusive.

### 3.1. Heavy Metals

Environmental contamination such as heavy metals whose source of release are mainly mining, industrial processing, and military use, may have a very wide impact, causing ecological, economic and consumer health consequences. For example, accumulation of heavy metals along the aquatic food chain in fishes is tissue specific [98,99,100]. Very often it occurs in fish muscles—an important source of nutrients—creating a risk for human health. For example, chronic consumption of methylmercury-contaminated (MeHg) fish leads to severe damage in humans, notably to the central nervous system [101].

Mercury is a highly bioaccumulative heavy metal, which contaminates the environment in both organic (e.g., methylmercury (MeHg)) and inorganic form. MeHg can easily cross the blood and placental barriers [102]. Its toxicity results from its high affinity with thiols. Due to this, it is able to bind with any protein containing cysteine or even methionine groups and disturbs cell functioning.

Histological and ultrastructural studies on adult zebrafish demonstrated that bioaccumulation of mercury leads to skeletal muscle damage. It is manifested by a decrease of the inter-bundle surface area, mitochondrial abnormalities such as changes in their shape and size, and cristae disorganization [53].

The effects of dietary MeHg exposure have also been assessed in adult zebrafish skeletal muscle at the molecular level by determination of gene expression [103]. MeHg affects the bioenergetics of adult zebrafish muscle via mitochondrial impairment. An environmentally relevant dose of MeHg inhibits state 3 mitochondrial respiration and cytochrome C oxidase (COX) activity leading to a decrease of ATP release in muscle cells without affecting their contractility [52]. In addition, other heavy metals have an impact on muscle mitochondrial bioenergetics [52]. Waterborne uranium at environmentally relevant concentrations (20–100 μg/L) also reduces mitochondrial respiration. The uranium acts through an increase in the inner mitochondrial membrane permeability and disruption of the transcriptional regulation of many respiratory genes [52,54]. Uranium exposure results in upregulation of the expression of the *COXI* and *ATP5F1* (a subunit of mitochondrial ATP synthase) genes, involved in mitochondrial metabolism [54]. In addition, uranium at a concentration of 20 μg/L leads to myofibrils and sarcomere disorganization [55].

Changes in skeletal muscle fiber organization in adult fish reflected by disruption of sarcomeric pattern and altered glycoprotein composition also occur after exposure to cadmium. The exposure to this heavy metal is also a cause of impaired muscular function associated with mitochondrial damage resulting in a reduction in swimming performance [56].

Cadmium shows a wide range of effects on fish muscle functioning. It leads to upregulation of different genes including protooncogenes [57], depletion of glycogen reserves and also interference with fiber excitation-contraction coupling [58,104]. During the zebrafish development exposure to cadmium can cause many toxic and teratogenic effects [105]. Toxic compounds, including heavy metals, especially affect incorrect trunk morphology in zebrafish [106]. Exposure to different toxicants can lead to the same phenotype, but their mechanism, time and place of action can vary. It was evidenced that cadmium exposure during zebrafish somitogenesis leads to disorderly packed somites that lose their typical chevron V-shape. Despite the fact that the myogenic regulatory transcription factors are expressed normally, loss of fast and slow muscle fibers occurs in the myotome. In cadmium-treated embryos primary and secondary motoneurons develop similarly to control embryos, whereas the axon growth is affected. The notochord, an organ essential for the patterning of the somites and the central nervous system, shows abnormal morphological features and fails to extend to the tail region [59].

Arsenic is found in the water systems due to both, natural geological processes and as a by-product of smelting, fossil fuel combustion, and pesticide production. Arsenic exposure can lead to a negative effect on human health by causing cancers as well as cardiovascular and neurological diseases. Arsenic exposure has also been shown to alter muscle growth and to cause developmental abnormalities [107,108]. The effect of arsenic exposure on developing zebrafish embryos depends on the concentration used in the experiment. The acceptable concentration of organic arsenic in drinking water is 10 µg/L (around 0.15 mM) therefore it is important to examine the impact of higher concentrations on organisms development [109]. At arsenic doses lower than 0.5 mM, no significant effects on embryos development were observed. Gradually, with increasing concentration above the initial dose, developmental abnormalities become more significant. Higher concentrations caused a reduction in embryo survival, and delayed hatching reduced growth and impairments in neural and cardiac systems [60]. The negative effect of arsenic exposure on muscle tissue has been confirmed in various studies. Arsenic-treated mice exhibit inhibited myogenic differentiation, suppressed skeletal muscle differentiation and impaired regeneration after injury [110]. Gaworecki et al. used killifish (*Fundulus heteroclitus*) as a model to examine the mechanisms by which the embryonic arsenic exposure can alter muscle development [111]. It has been demonstrated that in a dose-responsive manner, arsenic contamination results in transcriptional changes in genes involved in the cell cycle and ubiquitination processes. It was also evidenced that arsenic exposure during embryogenesis significantly reduces the average muscle fiber size and leads to aberrant muscle formation [111].

### 3.2. Organic Pollutants

Organic pollutants are a very diverse group of organic compounds exhibiting different effects. They may result from biodegradation of contaminants released into the environment. One of the greatest sources of organic pollutants contaminating fresh water are sewage effluents. Some of them such as EDCs (endocrine disrupting compounds) show peculiar properties. The EDC class of compounds encompasses diverse chemicals, which display a common functional feature consisting in the ability to, at certain doses, interfere with the endocrine system. This can be the cause of different disturbances including tumors and developmental disorders in which skeletal muscles are also affected.

#### 3.2.1. Endocrine Disrupting Compounds

Xenoestrogens or environmental estrogens can alter hormone signaling and due to this activity belong to the EDCs. They are regarded as serious environmental hazards that have an impact on both wildlife and humans. For monitoring xenoestrogens interfering with normal endocrine function, researchers take advantage of expression levels of some genes including genes coding for vitellogenin, the eggshell protein *zona radiata*, and cytochrome P450 aromatase [84]. However, this method is not sufficient for a comprehensive assessment of the effects of EDCs. The need to develop biosensors for xenoestrogens focusing not only on the reproductive system arose from the fact that xenoestrogens may also affect other body systems which have not been established previously as targets for estrogens in fish [112]. Recently, such biosensors based on a sensitive transgenic zebrafish model were successfully developed [92]. An estrogen-responsive transgenic zebrafish line has been generated allowing real-time assessment of effects of environmental and endogenous estrogens in a whole body system due to the specific expression of the GFP [92]. This highly sensitive biosensor enables both detection of a variety of EDCs at environmentally relevant concentrations and identification of target tissue or body system including muscles. EDCs similarly to other contaminants display a wide range of mode of action affecting different signaling pathways.

Bisphenol A (BPA), used to manufacture polycarbonates and epoxy resins, is a ubiquitous pollutant entering freshwater and marine systems and is also classified as an endocrine disruptor [113]. It interferes with a number of nuclear receptor pathways. At an ecologically relevant concentration (20 μg/L) it impairs swimming performance, heart rate, muscle and cardiac SERCA activity and gene expression in adult fish. It was reported that many of these responses are temperature-specific and non-monotonic [61].

Apart from the obvious link between dose and toxicity defined by Paracelsus “The dose makes the poison”, also more complex relationships depending on environmental factors such as temperature, which modulate toxicants’ mode of action, can be observed. It is particularly important in the context of global warming, since fish sensitivity to EDCs such as BPA is temperature-dependent. Research concerning this issue implies different possibilities. On the one hand, a temperature-inducible increase in dose-dependent BPA toxicity seems to be likely [61]. On the other hand, it does not exclude the inverse interaction, in which BPA exposure could compromise different abilities to tolerate new thermal environments. In considering these issues, it should be taken into account that the effects of BPA pollution are very dynamic, which results from its ability to interfere with a wide range of biological receptors including thyroid, estrogen and glucocorticoid receptors [114]. This is in agreement with the fact that thyroid hormone is important to maintain physiological performance reflected in skeletal muscle and cardiac responses at cold temperatures in adult zebrafish [115,116].

#### 3.2.2. Pesticides

Pesticides are a mixture of substances intended for preventing, destroying, repelling or mitigating any pest (insects, mites, nematodes, weeds, rats, etc.). Each year, nearly five billion tons of synthetic pesticides (including herbicides, insecticides, fungicides, etc.) are used globally [117,118]. Worldwide consumption of pesticides for agricultural use is constantly increasing from 0.49 kg/ha in 1961 to 2 kg/ha in 2004 [119]. Research carried out so far among different groups of pesticides demonstrates their negative impact on human health, in particular observed in fetuses, infants and children [120,121,122,123]. Many insecticides act mainly by disrupting the signaling mechanism in the central nervous system (CNS), thereby inhibiting neurological function [118]. Studies on mice and rats demonstrate that fungicides cause testicular, bladder transitional cell and hepatocellular tumors. Fungicides that penetrate the soil and subsequently enter the aquatic environment cause reproductive and developmental side effects in fresh water fish [124,125].

Because of the widespread use of pesticides and their uncontrolled release to the environment, it is necessary to examine their different side effects on living organisms. In understanding the mechanisms of disruption at the early stages of the developmental effects of pesticides in vertebrates, zebrafish is a very helpful model organism.

Organophosphorus (OP) compounds are a class of AChE inhibitors, which are applied, among other applications, as pesticides. The inhibition of AChE by OP compounds leads to accumulation of the neurotransmitter ACh at the cholinergic synaptic clefts, with the consequent long-term activation of the nicotinic and muscarinic ACh receptors (AChR) and overstimulation of cholinergic neurons as well as hyperexcitation and seizures. Following the initial cholinergic overstimulation, a cascade of downstream events occurs that lead to secondary neuronal muscle toxicity [63,126,127].

Previous studies in the *achesb55* and *zim* (*ache*) mutant zebrafish larvae have shown that a lack of functional AChE protein causes progressive myopathy [127,128]. To investigate whether application of OP compounds have the same influence on muscle development as in two *ache* mutants, zebrafish larvae were subjected to 300 nM CPO (chlorpyrifos-oxon) concentration. Morphological analysis was performed on embryos at 1–3 dpf (days post fertilization). These studies showed that, despite the fact that CPO exposure reduced the AChE activity, 87% of individuals from the experimental group exhibited morphological features similar to control embryos. The lower CPO stability in aqueous systems and chorion presence might explain this phenomenon [62].

Among the most commonly used OPs, chlorpyrifos (CPF) is extensively studied in the context of developmental neurotoxicity. Faria et al. exposed zebrafish larvae at 7 dpf for 24 h to different concentrations of the CPO compound. The authors found that the gradual increase of CPO concentration caused a decrease in AChE activity. Examined embryos showed a reduction in length of trunk and axial slow muscle fibers and changes in the structure and activity of muscles, including a reduction of motor activity. The highest CPO concentration led to complete embryo paralysis. These symptoms significantly mimicked many effects of OPs in humans [63].

Sodium metam (NaM), as an agricultural pesticide, belongs to the dithiocarbamate (DTC) chemical class. Zebrafish embryo exposed to DTC is characterized by distorted notochord accompanied by altered expression of notochord and muscle developmental markers [64,129]. Detailed analysis revealed that NaM specifically affects fast muscle development [66].

#### 3.2.3. Other Organic Pollutants

Polybrominated diphenyl ethers (PBDEs) are flame-retardants produced and used around the world. They belong to ubiquitous environmental pollutants detected in different media, including water, air, chicken eggs, fish, human blood and breast milk, raising a significant concern for human health public health [130]. Recently, it was found that long-term chronic exposure to a low dose (from 0.001 to 1 µM) of deca-BDE (BDE-209), a commercial PBDE mixture, affects both the parental generation and their offspring. In parental fish, deca-BDE leads to abnormalities in gonad development, male gamete quantity and quality and overall fitness. In the case of F1 offspring, delayed hatching and motor neuron development, loose muscle fiber and neuro-behavioral alterations including slow locomotion behavior in normal conditions and hyperactivity when subjected to light–dark photoperiod stimulation were observed [67].

4-Nonylphenol (4-NP) is a product of biodegradation of alkylethoxylates, which is a group of nonionic surfactants that are widely used in the manufacturing of detergents, plastics, paints, and cosmetics [131]. It is an estrogen-mimicking compound capable of disrupting endocrine signals and leading to developmental disorders in aquatic species [85].

4-NP affects notochord and muscle development, manifested in reduced motility and impaired swimming behavior in embryo [68]. The swimming abnormalities are accompanied by alteration in the expression levels of two neuroendocrine hormones: an increase in the case of the stress hormone CRH (corticotropin-releasing hormone) and a decrease in the case of LHB (luteinizing hormone beta). The most prominent effect of 4-NP action on muscle activity is an alteration in the relaxation mechanisms, which could explain the abnormal swimming pattern exhibited in larvae. However, the mechanism underlying this phenomenon remains elusive. As the authors suggest, the impairment may be connected with a lack of energy accumulation (ATP or ADP) resulting in cellular Ca^2+^ removal or a slow dissociation of the actomyosin complexes.

## 4. Drugs, Stimulants/Depressants, and Cosmetics

The invention of new drugs, stimulants and cosmetics is accompanied by the release of significant amounts of these compounds into the environment, which can bring unknown health risks. On the one hand, drugs and cosmetics are designed to be metabolically stable, retaining biological activity, but, on the other hand, in the case of their release, their stability may have a pernicious effect, causing a low degradation rate, which might potentiate environmental persistence.

### 4.1. Drugs

Many dangerous toxins, including insecticides, pesticides and chemical weapons, act by inhibiting AChE. It is worth noting also that many pharmacological therapies based on the use of compounds inhibit AChE, for example, treatment of the autoimmune disease myasthenia gravis, glaucoma, and Alzheimer’s disease [132]. However, AChE inhibitors induce overexpression of AChE-R in muscle and lead to altered neuromuscular structure in mice [133], suggesting the participation of ACh-R in the etiology of myopathic syndromes.

Behera et al. studied the application value of four drugs via a phenotypic comparison between the wild-type strain and a transgenic *ache* mutant zebrafish [69]. An important advantage of the zebrafish model in this experiment was a high resemblance to human AChE. Furthermore, *Danio rerio* is devoid of the additional acetylcholine-degrading enzyme [127]. The *ache* mutant provides phenotypic criteria—a total lack of ability to degrade acetylcholine—making it an objective standard to evaluate the effect of AChE inhibitors on the phenotype of the wild-type strain. In the experiment embryos were treated with four inhibitors, i.e., GAL (1,2,3,4,6,7,7a,11c-octahydro-9-methoxy-2-methylbenxzofuro [4,3,2-efg][2]benzazocin-6-ol), ESE (1′methylpyrrolidino [2′:3′:2:3] 1,3 dimethylindolin-5-yl N-methylcarbamate), TAC (1,2,3,4-tetrahydro-9-aminoacridine), and EDRO ([*3-hydroxyphenyl*] dimethylethylammonium bromide), which were applied from the zebrafish 5-somite stage onwards. Only one of them (GAL) provided an identical image of phenotypic changes to that observed for *ache* mutants. In the case of both the *ache* mutant and GAL-treated embryos, three common characteristics of the phenotype were observed: motility impairment induced by myopathy, no effect on the differentiation of neurons of the spinal cord, no changes in nACh-receptor (nicotinic acetylcholine receptor) activity. The other inhibitors exhibited additional impacts that were not seen in *ache* mutants. Two inhibitors, TAC and EDRO, caused a disturbance in the development of spinal cord neurons. Exposure to the inhibitor ESE gives similar effects to the genetic removal of nAChE-R in *ache* mutants—suppression of muscle defects [127]. This property of ESE implies that it worked as an antagonist nAChR.

Statins are the main pharmacological agents, used in the treatment of cardiovascular diseases. These substances have a well-proven record in reducing the cardiovascular mortality of patients with or without coronary artery diseases. The therapeutic effect of statins is based on reducing the level of low-density lipoprotein (LDL) cholesterol by competitively inhibiting an enzyme that catalyzes the endogenous synthesis of cholesterol in the liver: 3-hydroxy-3-methylglutaryl coenzyme A (HMG-CoA reductase). Although statins are well tolerated in most cases, they can induce myopathy in some patients, with a variety of clinical symptoms, such as mild pain, muscle weakness, and even fatal rhabdomyolysis [134,135]. In a clinical setting, a myopathy bioindicator is creatine phosphokinase (CPK), an enzyme which is released from disrupted myocytes. This enzyme is unstable, so its circulating level in blood can fall before blood sampling and lead to false negative results. Studies carried out by Shinh-Hao Huang et al. simultaneously confirmed the toxicity of statins and identified the novel diagnosis method of early stage statin-induced myopathy [70]. Zebrafish larvae exposed to statins exhibit distortion of the myosin filament and in consequence significantly shortened sarcomeres of skeletal muscles. The reduction or inhibition of the mevalonate biosynthetic pathway, caused by statins can be observed with second-harmonic generation (SHG) imaging as a microstructural abnormality. Moreover, the microstructural changes in the length of sarcomeres in a zebrafish model also demonstrated a strong correlation with the dosage and duration of treatment [70].

Clofibric acid (CA) 7 is an active metabolite of clofibrate—a peroxisome proliferator-activated receptor alpha (PPARα) which was used as a therapeutic agents lowering blood lipids. This compound, often found in the aquatic environment [136], is included among fibrates which regulate lipid metabolism [137]. It affects muscle through changing its composition. Chronic, lifetime exposure to CA induces in the F1 generation decreased growth and lower muscle triglyceride content and affects male gonad development [71].

Diclofenac (sodium 2-[2-(2,6-dichloroanilino)phenyl]acetate) is commonly used to relieve pain and inflammation. It is a member of non-steroidal anti-inflammatory drugs (NSAIDs), acting as an inhibitor of cyclooxygenase, an enzyme responsible for the synthesis of prostanoids. The exposure of zebrafish embryos to diclofenac results in various developmental defects including cardiovascular and muscle degeneration. The mechanism of diclofenac toxicity is based on interactions with macromolecules such as DNA, and induction of alteration of genes expression or the regulation of downstream genes [72]. Molecular pathways underlying its mode of action still remain elusive.

### 4.2. Cosmetics

Among the substances designed for beauty enhancement and protection containing ultraviolet (UV) filters are sun creams. They protect human skin from UVA and UVB radiation, but they also exert several side effects. Active compounds in UV filters can bind to hormone receptors, show hormonal activity and might have endocrine disruptive effects [138] (see Section 3.2.1). Due to their photo stability and lipophilicity, UV filters tend to accumulate not only in human skin but also in the aquatic environment [139].

A zebrafish model was used to test teratogenicity of 4-methylbenzylidene camphor (4-MBC) during early development [73]. 4-MBC is an active ingredient of UV filters and has the ability to penetrate human skin and tissue. After several applications of sun cream during the day, the 4-MBC was found in the plasma obtained from human blood samples [140]. 4-MBC shows toxicity due to estrogenic endocrine disruption and contributes to estrogen-mediated cancer occurrence in research conducted on MCF-7 breast cancer cells and in vivo in rats [141,142]. Li et al. reported that 15 µM 4-MBC exposure caused abnormal axial curvature, impaired tactile response, and immotility in zebrafish embryos. 4-MBC may affect somitogenesis, since the segmentation period is the most sensitive to exposure [73]. It was revealed that somites are altered and showed a distorted chevron shape. Moreover, the pattern of slow muscle fiber organization was affected. The studies led to the conclusion that 4-MBC has an acetylcholinesterase inhibitory effect, which affects normal slow muscle development and axon pathfinding, resulting in developmental defects in zebrafish embryos [73].

Recently Balázs’ [74] group examined the hormonal and cytotoxic activities of several UV filters, which were found in high concentration in water samples from Swiss lakes in the summer season [74,143]. The zebrafish embryos were used to test one of them, namely benzophenone-3 (BP-3). In experimental conditions BP-3 causes a lack of swim bladder inflation, which in higher concentrations causes fish mortality. Exposure to BP-3 leads to tail deformation. Moreover, malformation of the somites could be also observed. These effects reduce the hatching rate of zebrafish embryos. The described cytotoxic effects of BP-3 were not observed at lower concentrations. No mortality of embryo or adult zebrafish occurred after exposure to 600 mg/L of BP-3, although it was found to influences gene expression [144].

### 4.3. Stimulants/Depressants

#### 4.3.1. Ethanol

Ethanol has a severe influence on early human development, including cardiac and central nervous system abnormalities, abnormal craniofacial features, and general developmental and intellectual delays. The spectrum of these disorders is known as fetal alcohol syndrome (FAS) [145]. Serious effects of alcohol exposure on embryonic development were first described in 1973 [146] and provide an example of the impact of food and drinks on human health.

Zebrafish has been used as an animal model to study different aspects of alcohol influence on embryonic development [147,148,149,150,151,152]. Especially zebrafish embryos are very useful because its external development, which allows to precise control the ethanol concentration and time of exposure. Disadvantages of zebrafish model include a lack of maternal-embryo interaction during gestation, and relatively high doses of ethanol needed to induce changes in the zebrafish comparing to mammalian models [32]. Sylvain et al. [75,76], reported that also motor neurons, synaptic properties at the neuromuscular junction and muscle undergo abnormal development when zebrafish embryos were exposed to alcohol. Zebrafish embryos were exposed to ethanol in different concentrations (from 1% to 3% *v*/*v*) from 8 to 24 hpf. General morphological changes include deformities, higher mortality rate, delays in hatching and fewer bouts of swimming in response to touch. Morphological effects tend to increase in severity and frequency in a dose-dependent manner. The microscopic analysis showed that slow muscles displayed abnormalities such as a lack of segment division, altered angles between dorsal and ventral hemi-segments and smaller muscle fibers. Fish exposed to alcohol had shorter and narrower muscle fibers.

#### 4.3.2. Caffeine

According to data published by the International Organization of Coffee, the popularity of caffeine products is on the increase, and the consumption of coffee in 2015 was more than 9.1 million tons per year, about 7% higher than in 2012. Caffeine can have both positive and negative health effects. On the one hand, many studies have shown positive health effects such as lowering the risk of type 2 diabetes mellitus and obesity [153] and reducing the symptoms of Parkinson’s disease [154]. A number of adverse health effects of caffeine, including atrial fibrillation risk [155], faster heart rate and mixed effects on blood vessels [156,157], jittery and shaky phenotype headaches, sleep problems, and even physical addiction [158], were also confirmed. Moreover, caffeine can freely cross the placenta and affect the fetus. Maternal caffeine intake during pregnancy has been found to be associated with a reduction in birth weight [159] and even behavior problems [160].

Immunohistochemical methods were used to better understand the toxic effects of caffeine on the development of the neuromuscular system in zebrafish. Treated animals displayed disruption in neuromuscular junction development and abnormal neurotransmitter secretion. Skeletal muscles specific antibody (MF20) in caffeine-treated embryos exhibited an unequal staining pattern of MF20 in the whole somites and rounded somitic edges. In addition, shortening and misguided axons of motoneurons with accumulation of secretory vesicles in the terminal part of the axon were observed [77].

#### 4.3.3. Nicotine

The World Health Organization (WHO) defines nicotine addiction as one of the biggest public health threats. It is estimated that the tobacco epidemic kills six million people annually, of which more than 600,000 deaths concern non-smokers who are exposed to the harmful effects of tobacco smoke [161].

Nicotine has been shown to affect fetal development, leading finally to spontaneous abortion, low birth weight, and even sudden infant death or significant cognitive, intellectual, and behavioral deficiencies in offspring [162,163].

The toxic effects of nicotine exposure on the motor system are connected with the over-activation of skeletal muscle-specific AChRs leading to motor skill impairment, including paralysis.

Svoboda et al. [78] revealed, by using a GFP-expressing islet-1 transgenic line [164], that nicotine exposure impairs the response to tactile stimulation of zebrafish embryos, and also changes the swimming behavior. The influence of different nicotine concentrations (5, 15 and 30 µM) was correlated with GFP expression level. However, only the highest dosage of nicotine caused embryo paralysis at 42 hpf. Constant nicotine exposure sustained this effect, whereas its removal abolished it. Moreover, nicotine exposure leads to a delay of secondary moto-neuron development, and this retardation continues into larval developmental stages. Neuronal abnormalities also involved axon path-finding errors in embryonic and young zebrafish [78,79].

In addition, immunohistochemical studies on a non-transgenic zebrafish strain confirmed that nicotine delayed development of the dorsal axons arising from secondary motoneurons and impaired innervation of the musculature [80,81]. Of interest, nicotine-induced alteration in muscle development was not observed in a zebrafish mutant (*sofa potato*) which lacks muscle-specific AChRs [165,166].

## 5. Conclusions

Besides drugs, cosmetics and other man-made artificial compounds designed for direct application, there is an urgent need for monitoring and assessing the effects of various bioactive chemicals which can affect the environment and thereby also human health, e.g., through bioaccumulation. These substances are either intentionally synthesized to find application in, e.g., agriculture as pesticides, or an unwanted waste of human activity such as farm effluents. Regardless of their origin, they need to be treated as potential pollutants, and their use if possible should be preceded by careful evaluation of the risks and benefits.

The studies presented in this review demonstrate the possibility of zebrafish application in toxicological research. The availability of various mutant lines creates the possibility of advanced phenotype analysis and enables deep insight into the toxic effects and biological activity of exogenously delivered compounds. The studies conducted in this field improve our understanding of the toxic effects of chemical compounds on vertebrate neuromuscular system development and function and provide an opportunity for further studies, which will allow the development of toxicant antidotes to neutralize the whole or different aspects of their toxicity. Moreover, they determine the direction for the development of modern biosensors based on zebrafish transgenic lines with improved specificity and sensitivity of different chemicals’ detection.

## Figures and Tables

**Table 1:** Toxicants effects on the development and functioning of zebrafish skeletal muscle.

Toxicant	Examples	Effect	Reference
Heavy metals	MeHg (methylmercury)	Alternations in muscle bioenergetics. COX activity inhibitions leading to a decrease of ATP release in muscle	[52]
		Skeletal muscle damage	[53]
	U (uranium)	Increase in the permeability of the inner mitochondrial membrane and disturbance in transcriptional regulation of respiratory genes leads to decrease in mitochondrial respiration	[52,54]
		Upregulation of the *COXI* and *ATP5F1* genes expression	[54]
		Disorganization in myofibrils and sarcomeres	[55]
	Cd (cadmium)	Changes in skeletal muscle fibers organization, reflected in disruption of sarcomeric pattern, and glycoprotein composition	[56]
		Disturbance in mitochondrial function resulting in a reduction in swimming performance	[56]
		Upregulation of different genes including protooncogenes	[57]
		Depletion of glycogen reserves in muscles	[58]
		Affected motoneurons axons	[59]
		Abnormal morphological features and length of notochord	[59]
	Arsen	Reduction of survival and growth	[60]
Organic pollutants-endocrine disruptors	BPA (bisphenol A)	Impairment of swimming performance, disturbances in muscle activity and gene expression	[61]
Pesticides	CPO (chlorpyrifos-oxon)	Reduced AChE activity but without alternation in muscle development	[62]
	CPF (chlorpyrifos)	Trunk and axial slow muscle fibers length reduction	[63]
		Dose dependent effect: from reduction of locomotor activity to complete paralysis of axial muscles	[63]
	NaM (sodium metam)	Distorted notochord and altered expression of mRNA markers for notochord and muscle development	[64,65]
		Disturbances in fast muscle development	[66]
Other organic pollutants	PBDEs (polybrominated diphenyl ethers)	In F1 generation: delayed hatch and motor neuron development, loose muscle fibers and neurobehavior alternations	[67]
	4-NP (4-nonylphenol)	Affected notochord and muscle development manifested in reduced motility and impaired swimming behavior	[68]
		Alterations in the expression level of two hormones: increase of CRH and decrease of LHB	[68]
		Alterations in the muscle relaxation mechanisms	[68]
Drugs	GAL (galanthamine)	Motility impairment induced by myopathy	[69]
	Statins	Distortion of the myosin filaments leads to shortened sarcomeres in skeletal muscles	[70]
	CA (clofibric acid)	Reduction in growth and lower muscle triglyceride content in F1 generation	[71]
	Diclofenac	Muscle degeneration	[72]
Cosmetics	4-MBC (4-methylbenzyli-denecamphor)	Abnormal axial curvature, impaired tactile response and immobility	[73]
	BP-3 (benzophenone-3)	Deformation of the tail, malformations of the somites	[74]
Stimulants/depressants	Ethanol	Red muscles—lack of segment division, altered angles between dorsal and ventral hemi-segments and smaller muscle fibers	[75,76]
		Shorter and narrower muscle fibers	[75,76]
	Caffeine	Disruption in the neuromuscular junction development and abnormal neurotransmitter secretion	[77]
	Nicotine	Impaired response to tactile stimulation, and changes in the swimming behavior	[78]
		Delay of secondary moto-neuron development leads to impairment in the innervation of the musculature	[78,79,80,81]

Abbreviations: AChE, acetylocholinesterase; AChRs, nicotinic acetylcholine receptors; *ATP5F1*, a subunit of mitochondrial ATP synthase gene; COX, cytochrome c oxidase; CRH, corticotropin releasing hormone; LHB, luteinizing hormone b.
